# Supplementary material for: Experiences of women undergoing assisted reproductive technology in Ghana: A qualitative analysis of their experiences
Source: PLoS One. 2021 Aug 11;16(8):e0255957. doi: 10.1371/journal.pone.0255957 (PMC8357082; doi:10.1371/journal.pone.0255957)
Supplement: S1 Appendix — (DOCX) [file pone.0255957.s001.docx]

**S1 Appendix**

## Interview Guide

We are conducting an academic project work on the topic “**Experiences of women undergoing Assisted Reproductive Technology (ART) at the Finney Hospital and Fertility Centre, New Bortianor, Ghana”.** We would be grateful if you could spend 30 minutes of your time to answer these questions.

Information is required solely for academic purposes and strict confidentiality is assured.

**SECTION A: Demographic Information**

- Age
- Occupation
- Educational background
- Marital Status
- Do you live with your spouse?
- Have you conceived before?

**SECTION B:**

**Experiences of Women Undergoing the procedure**

1. How did you know about ART?
2. Why did you decide to go for ART?
3. Tell us what you know about ART?
4. Were you anxious about the treatment outcome?
5. How has ART impacted your life? (**Probe** further to know how it has affected their work pattern, social life, sexual life and others)
6. How will you describe the whole ART process? (**Probe** further to know if she has ever thought about giving up at a point)

**Challenges**

1. What challenges did you face during the procedure?
2. What were the challenges you faced in raising money for the procedure? (**PROBE** further to know how long it took to raise the money and the sacrifices she had to make)
3. Do you feel stigmatised as a woman resorting to ART to have child/children?
4. How has ART affected your marriage? **(PROBE** further to identify the problems in her marriage as a result of ART)

**Role of Significant Others**

1. Who were the people involved in your treatment period? (**PROBE** to know which people: spouse, family members)
2. How did their actions influence the procedure? (**PROBE** further to identify how the actions of nurses, husband, doctors, and family members **encouraged or discouraged** her)
