# Supplementary material for: Experiences of women undergoing assisted reproductive technology in Ghana: A qualitative analysis of their experiences
Source: PLoS One. 2021 Aug 11;16(8):e0255957. doi: 10.1371/journal.pone.0255957 (PMC8357082; doi:10.1371/journal.pone.0255957)
Supplement: Attachment — Submitted filename: Response to Reviewers.doc [file pone.0255957.s002.doc]

School of Basic and Biomedical Sciences

University of Health and Allied Sciences

PMB 31, Ho

Ghana – West Africa

July 2, 2021

Dear Academic Editor,

**RESPONSE TO REVIEWERS’ COMMENTS**

**Journal:**	PLOS ONE

*Manuscript ID:*	PONE-D-21-02324

**Title of Manuscript:**	Experiences of Women Undergoing Assisted Reproductive Technology in Ghana: A Qualitative Analysis of their Experiences

**Authors:**	Judith A. Anaman-Torgbor, Justice Wiston Amstrong Jonathan, Lily Asare, Bernice Osarfo, Rita Attivor, Afia Bonsu, Elizabeth A. E. Fialor and Elvis E. Tarkang.

Based on your decision on our manuscript titled, “Experiences of Women Undergoing Assisted Reproductive Technology in Ghana: A Qualitative Analysis of their Experiences”, which was communicated to us on May 24, 2021, we have considered the reviewers’ comments and have accordingly revised and incorporated changes into the revised version.

We believe that we have sufficiently addressed the issues raised by all the reviewers as well as the suggestions made by the Academic Editor, and therefore, we are hopeful that the revised manuscript would be acceptable to the journal for publication.

We would like to indicate that the order of authors as arranged on the manuscript is what has been agreed upon by all the authors.

Looking forward to hearing from you soon.

Thank you.

Yours sincerely,

Justice W. A. Jonathan

(Corresponding Author)

**EDITOR’S COMMENTS TO AUTHORS**

| **Reviewer’s Remarks** | **Authors’ Response** | **Reference Page** |
| --- | --- | --- |
| 1. Please ensure that your manuscript meets PLOS ONE's style requirements, including those for file naming. The PLOS ONE style templates can be found at | **Manuscript has been prepared in accordance with PLOS ONE’s style requirements.** |  |
| 2. When reporting the results of qualitative research, we suggest consulting the COREQ guidelines: <http://intqhc.oxfordjournals.org/content/19/6/349>. In this case, please consider including more information on the number of interviewers, their training and characteristics; and please provide the interview guide used.   Furthermore, in your Methods section, please provide additional information about the participant recruitment method and the demographic details of your participants. Please ensure you have provided sufficient details to replicate the analyses such as: 1)  a description of any inclusion/exclusion criteria that were applied to participant recruitment and 2) a description of how participants were recruited. | **The suggestion was considered and the needed information and document has been provided.**  **A description of the inclusion and exclusion criteria as well as the sampling strategy has been provided.** | Page 9  IDI Guide (S1 Appendix)  Pages8-9 |
| 3. Please ensure that you include a title page within your main document. We do appreciate that you have a title page document uploaded as a separate file, however, as per our author guidelines ([http://journals.plos.org/plosone/s/submission-guidelines#loc-title-page](http://journals.plos.org/plosone/s/submission-guidelines#loc-title-page)) we do require this to be part of the manuscript file itself and not uploaded separately.  Could you therefore please include the title page into the beginning of your manuscript file itself, listing all authors and affiliations. | **This information was considered in the original manuscript – a title page in the main document.** |  |
| 4. Your ethics statement should only appear in the Methods section of your manuscript. If your ethics statement is written in any section besides the Methods, please delete it from any other section. | **The suggestion was considered and the ethics statement on the title page has been deleted.** |  |
| 5. Please include captions for your Supporting Information files at the end of your manuscript, and update any in-text citations to match accordingly. Please see our Supporting Information guidelines for more information: http://journals.plos.org/plosone/s/supporting-information. | **Not applicable. No supporting information file included.** |  |

**Reviewer One**

| **Reviewer’s Remarks** | **Author’s Response** | **Reference Page** |
| --- | --- | --- |
| They should follow the correct steps to conduct a qualitative analysis. It should include reliable references upon which researchers have relied in applying the qualitative analysis.  They should Draft and organize the data. This could be transcribing the interview, organizing field notes from observations or ensuring all documents used in the analysis are available.  They should Categorize data into themes and code data elements and creating categories. | **The data analysis section has been revised accordingly. The revised section reads:**  **“The data were manually analysed using content analysis to identify patterns across the data sets [11, 12]. Content analysis was considered for this study because of its flexibility; it is applicable to a data-driven or theory-driven analysis. The analysis started with immersion into the data, involving verbatim transcriptions of all the audio recordings by the Principal Investigator and verified independently by the research team members. The team read the interview scripts multiple times in order to accurately capture the accounts of participants’ experiences. The transcripts and the field-notes taken during the interviews were reflected upon and coded. Key themes were identified from the different transcripts and displayed in matrices to enable systematic examination of similarities and patterns among various responses[12].** | Page 10-11 |
| They should Present the collected data | **All data related to this study have been included in the manuscript.** |  |
| There are many misspellings, using capital letters in the wrong places | **The misspellings captured have been corrected.** |  |
| The paper should be rearranged so that it matches the basic elements needed for the research | **The suggestion has been considered.** |  |
| The objectives of the research and its importance should be shown more clearly. The importance of the research has not been shown well | **The objective of the research and its importance have been addressed in the last two statements in on page 7 and in Table 1 in page 8 – statement of significance.** | Pages 7 and 8 |

**Reviewer Two**

| **Reviewer’s Remarks** | **Author’s Response** | **Reference Page** |
| --- | --- | --- |
| A brief discussion (in discussion part) on, what additional knowledge this study contributing to us. (As author mentioned in Table 1) | **The suggestion has been considered** | Page 19 |
| Three Themes of the study should be linked properly. As I see, there is no linkage between the Themes. | **Statements linking the study themes have been included in the main document** | Pages 16 and 17 |
| One additional section can be added to make this study far better. i.e. If author could mention some of the existing policies taken by government, and authors view point on policy recommendation | **An additional section has been included in the main document** | Page 22 |
|  |  |  |

Reviewer Three

| Introduction Page 4: Please add the source to the statement: “infertility is one of the reasons why some marriages end in divorce” | **The in-text citation has been provided on page 6 and full citation on page 22** | Page 6 & 22 |
| --- | --- | --- |
| Please specify the date of the prevalence rates (3.5% to 16.7% in developed nations and 6.9% to 9.3% in less developed countries) of infertility specify in the introduction. | **The date has been specified in the main document.** | Page 6 |
| Methodology  The abstract states that fifteen participants were invited and interviewed while in the methodology (page 7) mentions “A total of 32 women were invited to take part in the interview. However, 19 of them accepted the invitation and were successfully interviewed”. Kindly adjust | **The inconsistency has been corrected** | Abstract & page 9 |
| Kindly relabel, the session “Rigor and Ethical Considerations” to “Ethical considerations” | **The suggestion has been considered and session has been relabelled, “Ethical Considerations”.** | Page 11 |
| Were all interviews conducted and in English? Kindly specify and add detail if needed. | **All interviews were conducted in English and this has been indicated in the main document.** | Page 10 |
| It will be good to strengthen the introduction with statistics and information about the coverage and use of ART in SSA and in Ghana, in particular. The authors include the prevalence rates of infertile which is totally fine. It will also be good if the authors include in the introduction the synthesis of the key findings of studies included in the discussion. It will provide the readers with more insight and the state of the art before the discussion. | **To the best of our knowledge (study team) ART has not received much attention from the public and the government of Ghana as such there are paucity of studies on the topic area and policies to that effect in Ghana.** |  |
| For data analysis, I would suggest using, if possible, qualitative software data analysis. It will strengthened the robustness of the study. | **Not applicable to this study.** |  |
| The reader wants to know more about the study interviewers: were they health personnel or non-health practitioner researchers recruited outside health facility? Since the interviews took place at the health facility participants may only reported the positive experiences with health providers during the process. Kindly clarify this in the methodology and discuss, if possible, the extent to which it may affect participants’ declarations toward health practitioners providing ART services. I think this is important since it has been reported that the nature of the healthcare system play an important role ART access. | **This has been clarified in the method section.** |  |
| Page 14: last verbatim, second phrase, kindly remove “my” (a surplus word). | **“my” has been removed from the last verbatim second in page 14.** |  |
| Discussion  I think the discussion session may be improved by adding more studies from sub-Saharan Africa and removing studies outside Africa if possible. Only five studies were referred to for the discussion, among which only on from SSA (South Africa). I will suggest making the discussion more clearer by including the setting (name of the countries or areas) of the studies involve in the discussion. | **Very few studies on the topic area have been conducted in the African setting.**  **The study areas have been included in the discussion.** |  |
| All of the findings presented in the current studies were in agreement with past studies according to the discussion. I will suggest the author recall clearly what is new in their study (maybe the current study is among the first in Ghanaian setting, etc….). | **This suggestion has been taken care of in the review.** |  |
